# Reimagining Dementia Care: A Complex Intervention Systematic Review on Optimising Social Prescribing (SP) for People Living With Dementia (PLWD) in the United Kingdom

**DOI:** 10.1111/hex.70289

**Published:** 2025-05-12

**Authors:** Evie Papavasiliou, Jessica Marshall, Louise Allan, Katherine Bradbury, Chris Fox, Matthew Hawkes, Anne Irvine, Esme Moniz‐Cook, Aimee Pick, Marie Polley, Amy Rathbone, Joanne Reeve, Dame Louise Robinson, George Rook, Euan Sadler, Emma Wolverson, Sarah Walker, Jane Cross, SPLENDID Collaboration

**Affiliations:** ^1^ University of Leeds Leeds UK; ^2^ University of East Anglia Norfolk UK; ^3^ University of Exeter Exeter UK; ^4^ University of Southampton Southampton UK; ^5^ University of Hull Hull UK; ^6^ Newcastle University Newcastle upon Tyne UK; ^7^ Meaningful Measures Ltd. Bristol UK; ^8^ Hull York Medical School Hull UK; ^9^ University of West London London UK

**Keywords:** dementia care, health services, people living with dementia, primary care, social prescribing

## Abstract

**Introduction:**

Dementia is a complex medical condition that poses significant challenges to healthcare systems and support services. People living with dementia (PLWD) often face complex needs, exacerbated by social isolation and difficulty accessing support. Social prescribing (SP) has been increasingly integrated into the United Kingdom's National Health Service (NHS) as a means to connect individuals with non‐clinical services to address these challenges. However, current research provides limited detail on specific SP interventions tailored to dementia care, leaving gaps in understanding the targeted needs, participation drivers, effectiveness and potential benefits for PLWD.

**Methods:**

A complex intervention systematic review of SP in dementia care was performed in the United Kingdom using an iterative logic model approach. Six databases and grey literature were searched, supplemented by hand searching for reference lists of included studies. Results were screened in a two‐step process, followed by data extraction. Risk of bias was assessed using Gough's Evidence of Framework. Reporting was informed by the Preferred Reporting Items for Systematic Review and Meta‐Analysis (PRISMA‐CI) extension statement and checklist.

**Results:**

Forty‐nine studies, reporting on PLWD, met the inclusion criteria. Findings indicate that SP for PLWD in the United Kingdom is varied and lacks focus, reflecting the diverse demographics involved. Interventions encompass cognitive, educational, psychosocial, physical, community and complementary therapies, of inconsistent classification, with some being umbrella interventions and others standalone services. Provided by the NHS, charities and integrated services, SP involves a range of referrers and connectors. Finally, individual outcomes show benefits such as increased independence and improved mood, but challenges pertaining to suitability and logistical issues, whereas systemic outcomes include cost savings and better service delivery, despite high implementation costs.

**Conclusion:**

SP pathways for PLWD are varied, with success relying heavily on adequately resourced and trained connectors. While benefits extend beyond health improvements, further research is needed to assess long‐term impacts, refine mechanisms and standardise evaluation metrics for SP effectiveness in dementia care.

**Patient and Public Contributions:**

A PPI advisory group, consisting of a person living with dementia and a caregiver, was actively involved throughout the review process, providing insights into the review questions, the logic model, emerging findings and interpretation of results.

## 1 Introduction

### 1.1 Background and Rationale

Dementia poses a significant global health challenge, affecting 55 million people worldwide, projected to rise to 78 million by 2030 and 139 million by 2050 [1]. In the United Kingdom, approximately 944,000 people currently live with dementia, a number expected to exceed 1 million by 2030 [2]. People living with dementia (PLWD) and their families face complex medical, social and emotional needs, often exacerbated by social isolation and difficulties accessing timely support [3, 4]. These challenges bring considerable strain, necessitating a comprehensive support approach spanning medical, psychological and social care throughout the illness trajectory. Post‐diagnostic support (PDS), which aims to improve quality of life through integrated care [5], remains critical but is hindered by significant gaps and inequalities in service provision [6].

Social prescribing (SP) is a promising approach to enhance PDS, addressing the non‐clinical needs of PLWD and their families through community‐based interventions. SP connects individuals to non‐clinical services offered by community organisations, including activities such as arts, physical exercise and social clubs [7, 8]. These interventions promote social engagement, reduce loneliness and improve well‐being for both PLWD and their carers, while also easing pressure on traditional healthcare services [9, 10].

Despite these benefits, integrating SP into dementia care faces obstacles, including inconsistent referral processes, lack of standardised guidelines, insufficient funding and weak collaboration between healthcare providers and community organisations [11, 12, 13]. While there is growing evidence for SP's role in mental health and well‐being [14, 15], limited research explores its *application* in dementia care. Gaps remain in understanding SP interventions' effectiveness, uptake and health outcomes for PLWD [16, 17, 18, 19].

This highlights the need for a systematic review of SP as a complex intervention within the PDS framework, aimed at identifying best practices and overcoming barriers to effectively integrate SP into dementia care, ultimately improving outcomes for PLWD and their families.

### 1.2 Review Aims and Questions

This complex intervention systematic review (CISR) aims to identify, describe and explore how PLWD and/or their carers engage with SP interventions. By examining the mechanisms, processes and circumstances involved, the review seeks to inform future implementation strategies and improve dementia care outcomes.

The review addresses the following questions:

1. What SP interventions are currently available for PLWD and/or their carers in the United Kingdom?
2. To which PLWD and/or their carers are SP interventions being delivered?
3. What are the mechanisms (incl. services and agents) by which SP interventions for PLWD and/or their carers are being instigated?
4. What are the processes through which PLWD and/or their carers receive SP interventions?
5. For what reasons/circumstances do PLWD and/or their carers participate in SP interventions?
6. What are the effects of SP on (i) PLWD and/or their carers and (ii) dementia‐related healthcare, and how are these measured?

This paper specifically reports findings for PLWD, chosen due to the heterogeneity and breadth of evidence identified. This approach enables detailed exploration, interpretation and evaluation of results. A separate paper will address findings for carers. Together, these aim to optimise the use of SP in dementia care.

### 1.3 Operating Definition

For this CISR, SP is defined as ‘a means for trusted individuals in clinical and community settings to identify non‐medical, health‐related social needs and connect individuals to non‐clinical supports and services within the community by co‐producing a social prescription’, Muhl et al. [20]. This definition emphasises two core components: (1) the *connector*, a trusted individual who provides holistic support and a personalised care plan, and (2) the *co‐produced care plan*, developed in equal partnership to address non‐medical health‐related needs.

## 2 Methods

The review protocol was registered on the Prospective Register of Systematic Reviews (PROSPERO; CRD42023428625) on 16 June 2023. Detailed methods have been described elsewhere [21].

### 2.1 Data Sources

A comprehensive search was conducted across multiple electronic databases (MEDLINE, EMBASE, PsycINFO, CINAHL, Scopus and Cochrane/CENTRAL) and grey literature sources (EThOS and CORE) (see Table S1 in Supporting Information). Manual reference list searches of included papers further supplemented the search.

### 2.2 Inclusion and Exclusion Criteria

**Figure 1:**
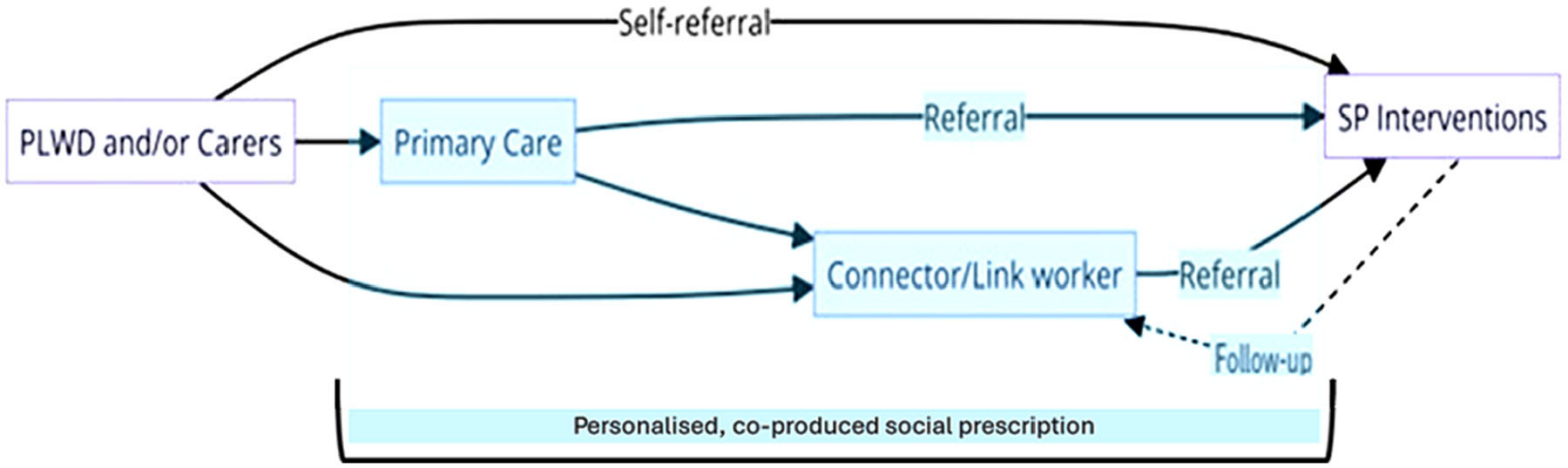
Social prescribing pathways.

Inclusion and exclusion criteria were based on the PICOTS framework [22], targeting studies involving individuals diagnosed with dementia or their caregivers engaged with core elements of the SP pathway in community settings (see Table S2 in Supporting Information). The inclusion/exclusion criteria reflected the core components of SP, as highlighted above, to define what should be classed as SP or not. The authors note that this led to the inclusion of studies not traditionally viewed as SP but that do contain its core components. To account for the complexity of SP and anticipated heterogeneity of evidence, inclusion/exclusion criteria were supplemented with a schematic representation of all possible SP pathways, Figure 1, developed to guide decisions on inclusion (adapted from Husk et al., 2020 [23]). This depiction served as a visual framework to accurately classify and include/exclude studies, mapping them against the various routes of SP, to ensure a consistent and comprehensive review process.

### 2.3 Search Strategy

The search strategy, developed in collaboration with a subject librarian, used relevant keywords and database‐specific terms (MeSH and Emtree) for SP and dementia. The search focused on UK‐based studies from 1 January 2003 to 15 June 2023, in English, without methodological restrictions. This time frame was selected to account for all research conducted in the past 20 years, considering that, while SP emerged as a concept in the late 1990s, the practice gained prominence in the early 2000s through pilot programmes and initiatives in various parts of the United Kingdom. As a result, earlier literature is less likely to provide relevant empirical evidence on structured SP interventions in dementia care. By setting this time frame, we ensure the inclusion of studies reflecting the modern evolution of SP while minimising the risk of incorporating outdated or less applicable findings.

### 2.4 Study Selection

After de‐duplication in EndNote V.20, citations were imported into Rayyan for screening [24]. Two reviewers (J.M. and S.W.) independently assessed titles, abstracts and full texts, with disagreements resolved by a third reviewer (E.P.) through consensus.

### 2.5 Data Extraction

Data extraction was conducted using a Microsoft Excel template designed to capture study characteristics and findings. J.M. and S.W. piloted data extraction on 10% of studies to ensure accuracy, with adjustments made as needed.

### 2.6 Quality Assessment

Study quality was assessed using Gough's Weight of Evidence (WoE) framework [25], scoring studies across three domains: coherence (WoE A), design appropriateness (WoE B) and focus relevance (WoE C). An overall score (WoE D) was calculated for each study. J.M. performed assessments, with 20% independently verified by S.W., showing high agreement.

### 2.7 Data Synthesis

A narrative synthesis [26] aligned data to the review questions, reporting results descriptively or thematically. The synthesis adhered to AHRQ and PRISMA guidelines for complex interventions [27, 28].

### 2.8 Logic Modelling and PPI

In this CISR, logic modelling was applied as an iterative framework to systematically map the relationships between key components of SP for PLWD. A process‐oriented logic model was developed using a structured six‐step approach, incorporating expert input, relevant theoretical frameworks and public involvement to ensure a comprehensive representation of how SP interventions operate within dementia care.

Logic modelling strengthened this review by structuring data extraction, analysis and synthesis in a way that accounted for intervention complexity. Specifically, it enabled the identification of causal pathways between intervention components, implementation factors and intended outcomes. This iterative approach allowed the model to evolve as new insights emerged [29, 30] from the included studies, ensuring that the synthesis process remained dynamic and reflective of real‐world SP applications.

The use of a colour‐coded logic model helped visually distinguish key elements and relationships at different stages of development, illustrating how different intervention components interacted over time. This structured approach provided a transparent method for understanding variation in SP delivery and outcomes across studies. The final iteration of the logic model is presented in Figure S1 (see Supporting Information). A detailed account of the logic model's development, significance and application in this review can be found in the published protocol [21]. A PPI advisory group, comprising a person living with dementia and a caregiver, provided input on the review questions, the logic model and emerging findings.

## 3 Results

**Figure 2:**
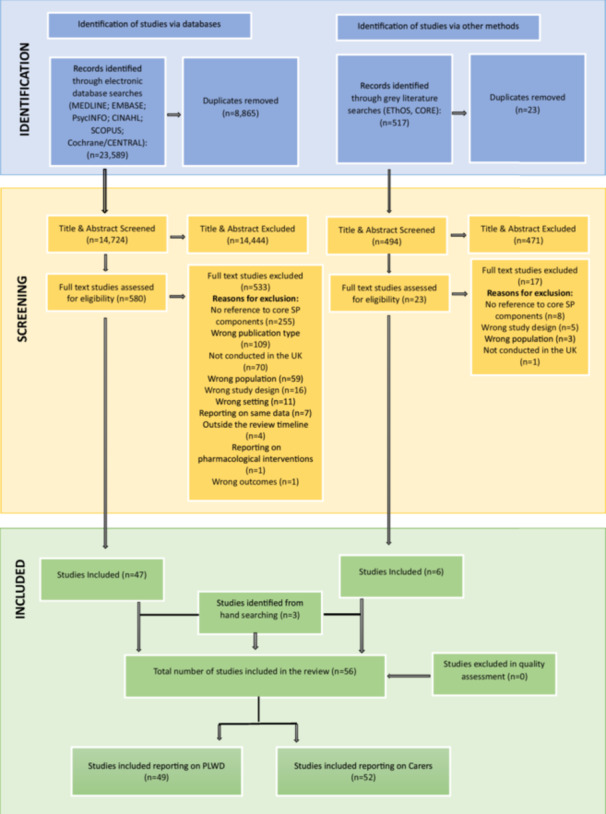
Preferred Reported Items for Systematic Reviews and Meta‐Analyses (PRISMA‐CI) flow chart.

Database searches retrieved 23,589 records. Following de‐duplication and title and abstract screening, 580 studies were assessed in full text for eligibility. Five hundred twenty‐nine studies were excluded. Six studies were included from grey literature searches after de‐duplication and full‐text screening of the 517 studies identified; three additional studies were included from the manual searching of reference lists of included studies. No studies were excluded during quality assessment. This resulted in 56 studies included in the review, 49 focused on PLWD, and these are reported in this first paper of the review series on SP in dementia care (see Figure 2).

### 3.1 Characteristics of Included Studies

The included studies comprised 39 original articles, 9 project reports and 1 PhD thesis, using qualitative (*n* = 31), quantitative (*n* = 5), mixed‐methods (*n* = 11) and RCT (*n* = 2) designs. All were conducted in the United Kingdom: UK‐wide (*n* = 6), England (*n* = 31), Scotland (*n* = 6), Northern Ireland (*n* = 1), Wales (*n* = 2), and England and Wales (*n* = 3) and were published between 2005 and 2023. Table 1 summarises the included studies.

**Table 1:** Characteristics of included studies.

**Author(s)**	**Year**	**Aims**	**Participants**	**Setting**	**Design and analysis**	**Weight of Evidence** A‐B‐C/D
Database searches						
Ahmed et al. [31]	2018	To examine staff roles and tasks in Community Mental Health Teams (CMHT) and memory clinics.	Informants^1^	England	Quantitative; descriptive statistics	2.83‐3‐2.75/2.86
Akhtar et al. [32]	2017	To report on the recommendations from interviewed family carers of PLWD on their experiences of using dementia cafés.	Carers	England	Qualitative; thematic analysis	2.75‐3‐2.75/2.83
Al‐Janabi et al. [33]	2020	To determine the mechanisms by which health and care services affect family carers' well‐being.	Carers and informants	United Kingdom	Qualitative; thematic analysis	3‐2.88‐2.75/2.88
Allward et al. [34]	2020	To evaluate the benefits of Cognitive Stimulation Therapy in supporting cognitive functioning for people with dementia.	PLWD	England	Quantitative; inferential statistics	2.92‐2.88‐2.88/2.89
Atcha [35]	2018	To identify the socio‐cultural issues in accessing dementia services in the population living in Blackburn with Darwen in the Northwest of England.	PLWD; carers and informants	England	Qualitative; thematic analysis	3‐2.88‐2.63/2.84
Baker and Irving [36]	2016	To analyse the operation of a pilot social prescribing scheme established collaboratively between a primary care trust (PCT) and community arts organisation (CAO), in the Northeast of England.	PLWD; carers and informants	England	Qualitative; thematic analysis	2.88‐2.88‐2.88/2.88
Bamford et al. [37]	2014	To test the transportability of a US case management model to primary care in England.	PLWD; carers and informants	England	Qualitative; thematic analysis	3‐3‐2.88/2.96
Bamford et al. [13]	2021	To identify the components of post‐diagnostic dementia support.	PLWD; carers and informants	England and Wales	Qualitative; thematic analysis	3‐3‐2.75/2.92
Bamford et al. [5]	2023	To develop an intervention to improve post‐diagnostic dementia care and support.	PLWD; carers and informants	England	Qualitative; framework analysis and realist evaluation	3‐2.88‐3/2.96
Brookes [38]	2017	To gather evidence to show whether Shared Lives could be a desirable service offer from the perspective of a carer or person with dementia and to support Shared Lives schemes to gain the confidence and skills they needed to be “dementia ready.”	Carers and informants	United Kingdom	Qualitative; thematic content analysis	2.96‐2.88‐2.75/2.86
Burgess et al. [39]	2021	To explore the experience of people with dementia, family carers and occupational therapists taking part in the COTiD‐UK intervention.	PLWD; carers and informants	United Kingdom	Qualitative; thematic analysis	2.79‐2.88‐2.75/2.81
Clarke et al. [40]	2013	To evaluate the Peer Support Networks and Dementia Advisors in the implementation of the National Dementia Strategy.	PLWD; carers and informants	England	Mixed‐methods; descriptive and inferential statistics; thematic analysis	2.92‐3‐2.88/2.93
Clarke et al. [41]	2018	To identify ways in which Dementia Advisors (DAs) and Peer Support Networks (PSNs) contribute to the well‐being and resilience of people with dementia and care partners.	PLWD; carers and informants	England	Mixed‐methods; descriptive and inferential statistics; content analysis	3‐3‐2.88/2.96
Cook [42]	2020	To investigate the meaning and use of urban woodlands and forests, and how they can contribute to the positive mental well‐being of people with dementia.	PLWD and carers	Scotland	Qualitative; thematic analysis	2.96‐2.88‐2.75/2.86
Eades et al. [43]	2018	To evaluate a community arts outreach intervention to establish how it engaged socially isolated people with dementia	PLWD and informants	England	Qualitative; thematic analysis	2.96‐3‐2.88/2.95
Femiola and Tilki [9]	2017	To understand the challenges faced by people with dementia and their carers and what they felt they needed for the Dementia Peer Support Project.	PLWD and carers	England	Qualitative; thematic analysis	2.83‐2.75‐2.75/2.78
Field et al. [44]	2019	To examine the acceptability of the intervention for participants in the United Kingdom and to inform its adaptation, before a randomised controlled trial.	PLWD and carers	England	Qualitative; thematic analysis	3‐3‐2.75/2.92
Field et al. [45]	2021	To explore and examine influences on the uptake of psychosocial interventions by people with early dementia after diagnosis.	PLWD; carers and informants	England	Qualitative; thematic analysis	3‐3‐2.88/2.96
Giebel et al. [46]	2021a	To explore the experiences of accessing post‐diagnostic dementia care for people living with dementia and carers both before and since the Covid‐19 pandemic and potential associated inequalities.	PLWD and carers	England	Qualitative; thematic analysis	2.92‐2.88‐3/2.93
Giebel et al. [47]	2021b	To evaluate a socially prescribed community service for PLWD and family carers.	PLWD and carers	England	Quantitative; descriptive and inferential statistics	3‐2.88‐2.88/2.92
Giebel et al. [48]	2021c	To explore potential health inequalities influencing care pathways for people living with dementia and their family carers.	PLWD and carers	England	Qualitative; thematic analysis	3‐3‐2.75/2.92
Górska et al. [49]	2016	To evaluate the impact of the pilot FGC service, delivered to people with dementia and their families, in terms of the experience of care provision by families and care professionals involved in the project.	Carers and informants	Scotland	Qualitative; thematic content analysis	3‐3‐2.75/2.92
Greenwood et al. [50]	2017	To investigate in‐depth informal carers’ experiences of attending cafés.	Carers	England	Qualitative; thematic analysis	3‐3‐2.88/2.96
Griffiths et al. [51]	2021	To understand the experiences of individuals with dementia or caring for someone with dementia, before and after a 12‐week relational counselling intervention.	PLWD and carers	United Kingdom	Qualitative; framework analysis	3‐3‐2.88/2.96
Griffiths et al. [52]	2022	To generate initial prospective theory building to develop a Dementia Support Worker intervention for PLWD and carers.	PLWD; carers and informants	United Kingdom	Qualitative; thematic analysis and realist evaluation	2.92‐3‐3/2.96
Hagan [53]	2020	To investigate the experiences of individuals recently diagnosed with dementia in Northern Ireland (NI) regarding how they were signposted to social support.	PLWD	Northern Ireland	Qualitative; grounded theory	2.83‐2.88‐2.75/2.82
Hewitt et al. [54]	2013	The aim of this preliminary project was to identify possible benefits of a structured group gardening programme for people with YOD.	PLWD and carers	England	Mixed‐methods; descriptive and inferential statistics; thematic analysis	2.92‐3‐2.75/2.89
Hoskins et al. [55]	2005	To evaluate the effectiveness of interventions provided by a Community Mental Health Team (CMHT) in reducing stress in carers of individuals with dementia.	PLWD and carers	Wales	Quantitative; descriptive and inferential statistics	2.92‐3‐3/2.97
Kelly and Innes [56]	2016	Reports the views of the project held by people newly diagnosed with dementia and their family members; as such it builds on the body of literature focusing on the views of people with dementia and their carers.	PLWD and carers	Scotland	Qualitative; thematic analysis	3‐3‐2.88/2.96
Killin et al. [57]	2018	To determine the feasibility of improving the quality of life of people with dementia (PWD) and their families with the DSP by adopting a qualitative approach, focusing on the needs of families recently diagnosed with dementia, the work they do to address these needs and how the DSP may have been used to this end.	PLWD and carers	Scotland	Qualitative; framework analysis	3‐3‐2.75/2.92
Levin et al. [58]	2018	To examine three interpretations of post‐diagnostic support (PDS) for dementia and to understand how best to support people recently diagnosed with dementia.	Informants	Scotland	Mixed‐methods; descriptive and inferential statistics; thematic framework analysis	2.96‐2.88‐2.75/2.86
Ling et al. [59]	2023	To explore the effect of providing ongoing support to people recently diagnosed with dementia and their carers.	PLWD; carers and informants	England	Qualitative; thematic analysis	2.96‐3‐2.88/2.95
Mac Rae et al. [60]	2022	To generate new evidence on the social impact of Dementia Friendly Walking Football (DFWF) that would inform the development of this activity within society and provide feasibility data to inform a future, more extensive research study.	PLWD; carers and informants	Scotland	Qualitative; thematic analysis	3‐3‐2.88/2.96
Maio et al. [61]	2016	To assess the effectiveness of the Admiral Nurses' approach from the perspective of family carers who had accessed their service to provide information for continuous improvement of practice, as well as providing evidence of users' satisfaction and effectiveness for commissioning purposes.	Carers	England	Quantitative; descriptive and inferential statistics	2.92‐2.88‐2.88/2.89
McDonald and Heath [62]	2008	To explore the provision of services for people with dementia and their carers in the three counties of Norfolk, Suffolk and Cambridgeshire in the area of the former Eastern Strategic Health Authority.	Carers and informants	England	Qualitative; thematic analysis	2.83‐2.88‐2.75/2.82
Mountain et al.^2^ [17]	2022	To determine the clinical effectiveness and cost‐effectiveness of an intervention to promote self‐management, independence and self‐efficacy in people with early‐stage dementia.	PLWD and carers	England	Randomised controlled trial	3‐3‐3/3
Piercy et al. [63]	2018	To report the evaluation of an integrated service, introduced as part of a local health and social care strategy to improve post‐diagnostic dementia care.	PLWD; carers and informants	England	Mixed‐methods; descriptive statistics and framework analysis	3‐3‐2.88/2.96
Prendergast et al. [64]	2022	To conduct interviews with stakeholders of a Shared Lives (SL) day support service to explore mechanisms and outcomes for the service.	PLWD; carers and informants	Wales	Qualitative; framework analysis	3‐3‐2.88/2.96
Sprange et al. [65]	2021	To identify the barriers and facilitators to the implementation of a complex psychosocial intervention through a study exploring the experiences of participants, carers and interventionists during a trial.	PLWD; carers and informants	England	Qualitative; framework analysis	2.92‐3‐3/2.97
Wenborn et al. [18]	2021	To estimate the clinical effectiveness of Community Occupational Therapy for people with dementia and family carers–UK version (Community Occupational Therapy in Dementia–UK version [COTiD‐UK]) relative to treatment as usual (TAU).	PLWD and carers	United Kingdom	Randomised controlled trial	3‐3‐3/3
Wheatley et al. [66]	2021	To examine common barriers to the delivery of PDS for dementia in England and Wales, including services from all sectors. We additionally describe a range of practical solutions used successfully by providers to address common barriers.	PLWD; carers and informants	England and Wales	Qualitative; thematic analysis	2.96‐3‐2.75/2.90
Wheeler et al. [67]	2015	To evaluate the Citizen Advice Bureau service provision, effectiveness and usefulness for service users.	PLWD; carers and informants	England	Mixed‐methods; descriptive statistics and thematic analysis	2.92‐2.75‐2.88/2.85
Willis et al. [68]	2009	To complete a qualitative investigation into the satisfaction with the service of those assessed and treated using the Croydon Memory Service Model (CMSM).	PLWD and carers	England	Qualitative; content analysis	2.96‐3‐2.88/2.95
Woods et al. [19]	2012	To assess the effectiveness and cost‐effectiveness of joint reminiscence groups for people with dementia and their family caregivers as compared with usual care.	PLWD and carers	England and Wales	Randomised controlled trial	3‐3‐3/3
Grey literature						
Ahmed et al. [69]	2017	To improve access to dementia services for BME communities in Salford; increase carer identification and registration; raise awareness of the needs of Salford's diverse communities and increase staff knowledge/develop evidence‐based decision‐making relating to minority communities who may access dementia services/general health and social care‐related services in Salford.	Informants	England	Qualitative; thematic analysis	2.83‐2.75‐2.75/2.78
Dayson et al. [70]	2014	To assess the impact of the pilot for its key stakeholders; to assess whether the aims and outcomes of the project had been achieved; to provide analysis of costs–benefits and return on investment, including assessing the cost savings and efficiencies to the NHS; to assess the effectiveness of the service delivery model and to establish a business case for future funding	Service users (incl. PLWD)	England	Mixed‐methods; descriptive and inferential statistics; thematic analysis	2.83‐2.88‐2.75/2.82
Dayson et al. [71]	2016	To evaluate the social and economic impact of the Rotherham Social Prescribing Service for people with long‐term health conditions	Service users (incl. PLWD)	England	Mixed‐methods; descriptive and inferential statistics; thematic analysis	2.88‐2.75‐2.75/2.79
Goodman et al. [72]	2019	To identify whether dementia‐friendly communities (DFCs) support people living with dementia and their carers to maintain their independence and feel valued as members of their local community, and, if so, which approaches have worked best and at what cost for which groups of people.	PLWD; carers and informants	England	Mixed‐methods; descriptive and inferential statistics; thematic analysis	2.88‐2.75‐2.75/2.79
Palmer et al. [7]	2017	To evaluate the benefits and limitations of a social prescribing pilot which took place in the Clocktower locality (London Borough of Bexley) over a 24‐month period, and this study forms the main body of the study.	Service users (Incl. PLWD and carers)	England	Mixed‐methods; descriptive and inferential statistics; thematic and narrative analyses	2.75‐2.75‐2.75/2.75

^1^ Individuals who provide supplementary or corroborative information about others or situations based on their observations, interactions and knowledge, offering insights and data from their own perspective and thereby providing a broader understanding and context about the primary subjects.

^2^ Data from the qualitative study embedded in the randomised controlled trial, as detailed in the report, were extracted from the paper by Sprange et al. [65], which focused solely on qualitative findings and preceded the report by Mountain et al. [17].

### 3.2 Heterogeneity, Focus and Nature of Available Evidence

The included studies exhibited significant heterogeneity in terms of design, ranging from cross‐sectional surveys and ethnographic observations to randomised controlled trials with diverse outcome measures. Variation was also evident across the PICOTS framework, including patient demographics, intervention types, comparison groups and outcome measures.

The studies varied in focus and the type of evidence reported, particularly:

1. *Adherence to the SP pathway*: Most studies did not report all pathway elements. However, studies were included if they addressed core components—connector, personalised care plan and engagement with a non‐clinical service or activity.
2. *Nature of evidence*: Some studies relied on carers and/or informants providing indirect or supplementary insights on PLWD, while others generalised findings across a broader range of services or populations that included PLWD.

Due to data heterogeneity, results are presented narratively, either descriptively or thematically, depending on the available evidence. Reporting follows the structure of the final iteration of the logic model used to guide data synthesis covering participants, interventions/services, mechanisms, processes, reasons/circumstances and outcomes.

### 3.3 Participants

In terms of participant characteristics, gender information was provided in 23 of the included studies, with 17 studies reporting exclusively [35, 42, 60] or predominantly [13, 17, 18, 19, 34, 39, 44, 47, 48, 53, 56, 57, 65, 68] on male participants. Age was reported in 22 of the included studies, with diverse samples of PLWD being enrolled, ranging in terms of age from early 40s/≤ 45 [54] to late 90s/≥ 95 [18, 19, 34, 43, 51]. Various dementia types, including Alzheimer's disease (AD), vascular dementia, Lewy body dementia, frontotemporal dementia, mixed dementia and dementia in Parkinson's disease, were addressed in 16 of the included studies, with 14 reporting three types of dementia or more [13, 17, 18, 19, 34, 42, 44, 45, 46, 47, 54, 63, 65, 68]. Information about time of dementia development, time of diagnosis and stage or severity was provided in 17 of the included studies, with 7 reporting on people with young onset dementia [13, 46, 48, 53, 54, 67], 2 reporting newly diagnosed cases [56, 59] and 8 people with early stage [17, 35, 45], mild to moderate dementia [19, 40, 44, 52], or dementia that had progressed to varying degrees [60]. Finally, 7 studies reported on living arrangements, including people living alone, maintaining full independence, or living in a care home, being semi‐independent and people cohabiting/living with family, reflecting the diverse living arrangements in this population [13, 17, 18, 36, 45, 60, 65].

### 3.4 Interventions/Services

45 studies reported on SP interventions for PLWD [5, 7, 9, 13, 17, 18, 19, 32, 33, 34, 35, 36, 38, 39, 40, 41, 42, 43, 44, 45, 46, 47, 48, 49, 50, 51, 53, 54, 56, 57, 58, 59, 60, 61, 62, 63, 64, 65, 66, 67, 68, 69, 70, 71, 72]. There were predominantly umbrella interventions comprising a wide range of activities, including: *psychosocial interventions*, the most frequently identified interventions, featuring dementia cafés, support groups and peer support networks [7, 9, 13, 17, 32, 33, 35, 40, 41, 45, 46, 48, 50, 51, 53, 56, 60, 65, 69, 70, 71, 72]; *cognitive interventions*, such as Cognitive Stimulation Therapy (CST) and memory clinics [19, 34, 35, 68]; *educational interventions* like post‐diagnosis courses and psycho‐educational programmes [48, 49, 58]; *case management interventions* involving tailored PDS [5, 13, 67]; *physical and well‐being interventions* including exercise programmes and well‐being activities [42, 47, 54, 61]; *community and social support interventions* involving practical support and shared lives schemes [36, 38, 43, 62, 64, 71]*; occupational and c*omplementary therapies *interventions* [18, 33, 39, 44]; and *digital and technology interventions* [13, 57]. A vast array of activities were identified within these umbrella interventions, ranging from arts, singing and dancing to exercise, tai chi, games, outings, aromatherapy and acupuncture. Findings, however, also demonstrated that some interventions (e.g., dementia cafes) classified as umbrella interventions in certain studies [7, 32, 33, 35, 50] appeared as activities within different umbrella interventions in other studies [56, 70]. Finally, the same umbrella interventions (e.g., dementia cafes) were found to encompass diverse activities in different studies such as arts‐based activities, singing, exercise, access to resources and services [32] or music, singing, quizzes, gardening, information provision, peer support and signposting to other services [50], thereby highlighting the variability of SP interventions for PLWD. Intervention components (e.g., individualised support or pair or group sessions), frequency/duration (e.g., short term vs. long term, weekly or monthly) and mode of delivery (e.g., online or in person) varied considerably across studies.

### 3.5 Mechanisms

41 studies reported mechanisms through which SP services for PLWD were instigated, provided or commissioned [5, 7, 9, 13, 17, 18, 19, 33, 34, 36, 38, 39, 40, 41, 42, 43, 44, 45, 46, 47, 48, 49, 50, 51, 53, 54, 55, 56, 57, 58, 59, 60, 61, 62, 63, 65, 66, 67, 68, 71, 72], with *public sector organisations* including NHS services and local authorities [5, 9, 17, 18, 19, 34, 36, 38, 39, 44, 45, 49, 56, 58, 65, 68] being the most prevalent, followed by *charitable and voluntary sector organisations*, such as Age UK VCSE, Alzheimer Scotland and faith‐based community organisations [7, 17, 33, 42, 46, 50, 51, 53, 54, 59, 60, 62, 71, 72] and *integrated services* featuring collaborative efforts amongst primary healthcare services, charities, local government, community services and/or academic institutions [13, 40, 43, 46, 47, 55, 57, 61, 63, 66, 67]. This extensive participation and wide‐ranging involvement of diverse stakeholders indicate a comprehensive strategy leveraging varied expertise and resources to support and enhance care for PLWD. A diverse range of mechanisms, through which SP services for PLWD were delivered, was identified in 36 studies [5, 9, 13, 17, 18, 19, 32, 33, 34, 36, 38, 39, 40, 41, 42, 43, 44, 47, 49, 50, 51, 53, 54, 55, 56, 57, 58, 59, 60, 61, 62, 64, 65, 66, 67, 68]. Such mechanisms comprised: *clinical staff* and *specialised therapists* such as occupational therapists, nurses, clinical psychologists, community psychiatric nurses, physiotherapists and horticultural therapists [5, 17, 18, 19, 33, 34, 39, 40, 41, 54, 56, 60, 61, 65, 68]; *non‐clinical staff* and *staff from charitable organisations* including café coordinators, volunteer coordinators, dementia navigators, group facilitators, Alzheimer Scotland staff and branch workers [9, 19, 32, 36, 38, 41, 42, 43, 47, 49, 50, 51, 53, 54, 56, 59, 60, 62, 64, 66]; and *other professional staff*, with their speciality or role not being specified [58, 67].

### 3.6 Processes

42 studies reported who instigated the pathway to SP interventions for PLWD, indicating a diverse range of stakeholders involved [5, 7, 9, 13, 17, 18, 19, 31, 32, 34, 35, 36, 38, 39, 40, 41, 42, 43, 44, 45, 46, 47, 49, 51, 52, 53, 54, 55, 56, 57, 59, 62, 63, 64, 65, 66, 67, 68, 69, 70, 71, 72]. Referrals were predominantly instigated by *primary care* including GPs, admiral nurses, dementia navigators based in primary care settings and community care duty services [5, 7, 13, 17, 18, 31, 35, 36, 40, 41, 47, 49, 51, 52, 53, 55, 59, 64, 65, 66, 68, 69]; *secondary care* including clinicians within NHS memory services, secondary care‐led enhanced memory assessment services and community mental health services [9, 13, 17, 18, 34, 39, 40, 41, 44, 47, 52, 53, 54, 55, 56, 59, 65, 67, 68, 69, 72] and *charities and voluntary sector organisations* including Alzheimer Scotland and Age UK Camden [7, 9, 17, 18, 19, 38, 40, 41, 42, 43, 44, 46, 47, 54, 57, 59, 62, 65, 67, 70, 71]. There were also *self‐referrals* via diverse routes and *family referrals* identified [9, 17, 18, 19, 40, 41, 53, 57, 59, 63, 64, 65, 67], highlighting the wide‐ranging involvement in the referral process.

Similarly, in 42 studies, a diverse range of individuals who connected PLWD to SP interventions were identified [5, 7, 9, 13, 17, 18, 19, 31, 34, 35, 36, 37, 38, 39, 40, 41, 42, 43, 44, 45, 46, 47, 48, 49, 50, 52, 53, 55, 56, 57, 58, 60, 62, 63, 64, 65, 66, 67, 68, 70, 71, 72]. These connectors included *clinical staff* from various roles and specialties, such as clinical dementia leads, admiral nurses, link workers, well‐being practitioners and social prescribers [13, 38, 42, 43, 45, 49, 50, 52, 56, 57, 60, 62, 64, 67, 72]; *staff from memory clinics* and *mental health teams* [31, 35, 66, 68]; *multidisciplinary teams* comprising staff from diverse specialties [34, 66]; and *personnel from third sector and community‐based organisations*, including befrienders and peer support network dementia advisors, were also identified as connectors [13, 38, 42, 43, 45, 49, 50, 52, 53, 57, 60, 62, 64, 67, 72]. Research teams, particularly in studies assessing the effectiveness of SP interventions, played a significant role in facilitating these connections [17, 18, 19, 65]. Inconsistency in the terms used to describe connectors was observed across included studies, with a variety of terms like link workers or link and support workers, social prescribers, dementia navigators or dementia care navigators and boundary spanners [7, 9, 13, 36, 42, 46, 47, 48, 52, 53, 58, 66] being used interchangeably across sectors, with their roles not being explicitly described and their place in the SP pathway not being explicitly determined/established.

### 3.7 Reasons/Circumstances

Of the 49 included studies, 29 reported reasons for (facilitators) and against (barriers) participating in SP interventions [9, 13, 35, 36, 37, 38, 39, 40, 41, 42, 43, 44, 45, 46, 47, 48, 49, 50, 51, 52, 53, 56, 63, 64, 65, 67, 69, 70, 72] (see Table 2). The primary reasons for participating in SP interventions included seeking emotional support, practical assistance, social engagement and coping strategies. *Emotional support* themes encompass the importance of being heard, expressing hopes and fears, and receiving support early and mental stimulation [9, 39, 43, 45, 72]. *Practical support* is highlighted as tailored advice, assistance with complex forms, and having a single contact point for guidance [40, 56, 65, 67, 69, 70]. *Social and community* aspects involve socialisation, interaction with others and being part of the community [36, 41, 42, 47]. Additionally, *adjusting to dementia* includes coping with symptoms, increasing activity levels and encouragement from relatives [44, 51]. *Knowledge and empowerment* are derived from early support following diagnosis, feeling empowered through information and interacting with peers/being with others with dementia [48, 49, 50]. *Trust and reliability* in the support workers and a supportive atmosphere were also crucial [38, 40, 52, 63]. *Activity engagement* includes enjoying remaining skills and engaging in purposeful activities [9, 64], while *shared knowledge* and experience focus on educating the community and interacting with peers [9, 41].

**Table 2:** Reasons for (facilitators) and against (barriers) participating in SP interventions.

	Theme	Sub‐theme
Reasons FOR	Emotional support	Importance of being heard and valued Expressing hopes and fears in a safe space Mental stimulation Opportunity for personal time Early support
	Practical support	Advice and signposting tailored to needs Assistance with complex forms (e.g., Disability Living Allowance) Single contact point Tailored support
	Social and community	Socialisation Interaction with others Being part of the community Trying new activities Educating the community Staying connected with the community
	Adjustment and coping	Coping with symptoms Increasing activity levels Developing coping mechanisms Participating in meaningful activities Encouragement from relatives
	Knowledge and empowerment	Early support following diagnosis Practical advice and support Feeling empowered by information—person‐centred approach
	Trust and reliability	Support worker as a point of trust Supportive atmosphere Open referral system Companions offering personalised support
	Activity engagement	Enjoying remaining skills Engaging in purposeful activities Flexible and personalised activities Multisensory activities Promoting independence
	Shared knowledge and experience	Sharing knowledge to educate the community Being with others with dementia—interacting with peers Supporting valued activities (e.g., maintaining interests)
Reasons AGAINST	Lack of cultural sensitivity	Services lack cultural sensitivity Stigma in specific cultural communities Need more tailoring to BME needs Different advertising roots
	Overwhelming information	Overwhelmed by advice and information Too many organisations acting as gatekeepers
	Transport issues	Lack of transport Transport difficulties (poor public transport) Geographical inequities Associated costs of food and travel
	Health and physical barriers	Physical health limitations Physical impairments, confusion and disorientation Sensory impairment
	Awareness, timing and access	Not being aware that services are available Need time to adjust to the diagnosis Untimely provision of support—intervention offered too soon Lack of accessible information and support Hard to access services without family support
	Inappropriate activities	Activities not suitable Preference for non‐dementia‐related activities Support groups not appealing
	Financial concerns	Cost of interventions Financial costs
	Psychosocial and emotional issues	Feeling like a burden to carers Embarrassment and anxiety Dementia stigma Denial of dementia symptoms Denial of diagnosis Anxiety about the future Fear and distress from seeing others further in the dementia journey Anxious about mixing with other people with dementia Gender imbalance in groups
	Communication issues	Poor communication between services Lack of consistent secondary healthcare support
	Practical barriers	Practical help over signposting Long waiting lists and limited availability Lack of referrals from primary care Postcode lottery of services Insufficient resources

Significant barriers preventing PLWD from participating in SP interventions included: *lack of cultural sensitivity*, where services were perceived as not tailored to meet the specific cultural needs of Black and Minority Ethnic (BME) communities, exacerbating stigma and limiting engagement [9, 13, 35, 40, 44, 45, 69, 72]; *overwhelming information*, with many individuals feeling burdened by the volume of advice and the gatekeeping roles of multiple organisations, making navigation of services complex [37, 38, 39]; *transport issues*, particularly the lack of available transport options, geographical inequities and associated costs, which hindered access to interventions [9, 13, 40, 45, 46, 47, 48, 52, 56]; *health and physical barriers* such as physical impairments and sensory difficulties [39, 42], making participation challenging; *awareness, access and timing* where individuals were not aware of services available, could not access them without family support or felt that interventions were sometimes offered too soon after diagnosis, not allowing them adequate time to adjust [42, 45, 46, 53, 69]; and *inappropriate activities* [13, 42, 43, 45, 46, 48]. Some PLWD also reported *psychosocial and emotional issues*, such as feeling like a burden to carers or experiencing embarrassment due to dementia‐related stigma, anxiety about mixing with other people with dementia or fear and distress from seeing others further in the dementia journey [9, 44, 45, 48, 51, 52, 56], while *financial concerns* regarding the cost of interventions added another layer of difficulty [46, 52, 69]. Finally, *communication issues* highlighted the frustration caused by inconsistent communication between services and a lack of referral support from primary and secondary healthcare providers [46, 48, 72] and practical barriers such as help over signposting, long waiting lists and limited availability, lack of referrals and insufficient resources [9, 13, 39, 46, 53, 69]. These themes emphasise the need for more accessible, culturally sensitive and coordinated approaches to support PLWD effectively.

### 3.8 Outcomes

Positive and negative outcomes from participation in SP interventions were identified in 39 studies indicating strengths, challenges and areas for concern [7, 9, 13, 17, 18, 19, 33, 34, 35, 36, 37, 39, 40, 41, 42, 43, 44, 45, 46, 47, 48, 49, 50, 51, 52, 54, 56, 57, 59, 60, 61, 63, 64, 65, 66, 67, 68, 71, 72] (see Table 3). *Enhanced independence* is observed through increased skills and self‐management techniques, which contribute to maintaining a sense of control and independence in daily life [13, 33, 41, 65]. *Improved mood and well‐being* are suggested by enhanced mood, reduced anxiety and better coping with symptoms [34, 44, 45, 59, 60, 64, 71]. *Social connectedness* shows a reduction in isolation and opportunities for socialisation and peer support, fostering a sense of community [7, 9, 35, 36, 40, 42, 45, 46, 47, 50, 56, 64, 72]. *Mental and cognitive benefits* are identified in improved cognitive functioning and reminiscence of cultural experiences [34, 43, 54, 60]. *Empowerment and identity* suggest that PLWD maintain a sense of purpose and self‐worth [13, 41, 42, 54]. *Practical support and resources* highlight continuity of care and better access to services, enhancing financial stability [37, 63, 67]. *Quality of life improvements* are concluded from increased engagement in meaningful activities and overall better well‐being [17, 64, 67]. *Positive relationships* show reduced burden and enhanced interactions with family and peers [7, 52], while *acceptance and adjustment* facilitate better coping with the diagnosis and decision‐making for the future [48, 51, 59]. Finally, *security and comfort* are observed through consistent support and a sense of safety [37, 68].

**Table 3:** Positive and negative outcomes from participation in SP interventions.

	**Theme**	**Sub‐theme**
Positive outcomes	Enhanced independence	Increased skills and self‐management techniques Independence in daily life and maintaining a sense of control
	Improved mood and well‐being	Enhanced mood and reduced anxiety Increased optimism and relaxation Better coping with symptoms
	Social connectedness	Sense of community and reduced loneliness/isolation Opportunities for socialisation and peer support Building new social networks
	Mental and cognitive benefits	Improved cognitive functioning Clearer thinking Reminiscence of childhood memories and cultural experiences
	Empowerment and identity	Maintaining a sense of identity and purpose Feeling valued and useful Enhanced self‐esteem and self‐worth
	Practical support and resources	Continuity of care Timely support and improved access to services Enhanced financial stability
	Quality of life improvements	Positive impact on daily life and well‐being Increased engagement in meaningful activities Improved quality of life
	Positive relationships	Enhanced relationships with family and friends/reduced burden Peer support and shared experiences Valued interactions with others
	Acceptance and adjustment	Help facilitate acceptance of the diagnosis Practical and psychological impact Feeling more positive about the future/better decision‐making for the future
	Security and comfort	Feeling of security and comfort Practical benefits of consistent and ongoing support
Negative outcomes	Intervention suitability	Disliking the session content Activities not appealing, enjoyable or helpful Lack of confidence with technology Cognitive and physical impairments limiting engagement Some activities evoke frustration or anxiety Loss of social contact or friendships post‐intervention
	Emotional impact	Anxiety from the evaluation process Anxiety about the future Fear of rejection Feeling disappointed with unmet expectations
	Service issues	Unclear service scope Untimely provision of support Confusion about the point of contact Inconsistent quality of care reviews Staff nervousness and insensitivity Assessment process causing stress and anxiety Limited duration of peer support Interventions coming too late
	Activity relevance	Activities not matching interests and hobbies Dependency on peer support due to a lack of information about other groups Resistance to using digital solutions due to a lack of confidence or apathy
	Social dynamics	Success dependent on PLWD/carer relationship Pre‐existing family dynamics affecting commitment Personal choice not respected enough
	Logistical challenges	Getting lost in venue areas Need for more signs or people to guide
	Outcomes	Outcomes (e.g., activities of daily living, cognition, quality of life and mood) either not significant or not maintained Disappointment that post‐intervention friendships did not continue Some activities considered pointless when they could be done at home

For PLWD, *intervention suitability* shows that some participants disliked session content, found activities either not appealing or not useful, and faced cognitive and physical impairments that limited their engagement, leading to frustration or anxiety [34, 40, 43, 45, 57, 61, 65]. *Emotional impact* reveals anxiety from the evaluation process, fear of rejection and disappointment with unmet expectations [17, 36]. *Service issues* highlight unclear service scope, inconsistent quality of care reviews and stress caused by the assessment process [37, 66, 68]. *Activity relevance* suggests that activities often did not match interests and hobbies, and there was a dependency on peer support due to a lack of information about other groups [40, 45, 46, 68]. *Social dynamics* indicate that success was heavily dependent on the relationship between PLWD and their carers, with pre‐existing family dynamics affecting commitment [39, 49]. Finally, *logistical challenges* include difficulties navigating venues and a need for more guidance, whereas *outcomes* show that improvements in various outcomes (activities of daily living, cognition and quality of life) were either not significant or not maintained, and there was disappointment that post‐intervention friendships did not continue [17, 18, 19, 60].

Finally, only eight of the included studies reported on how outcome measures for PLWD were assessed [17, 18, 19, 34, 40, 41, 47, 54], indicating varying assessments across several domains. These domains included: *mental and psychological well‐being*, assessed by the Short Warwick‐Edinburgh Mental Well‐being Scale and Diener's Flourishing Scale [17, 34, 47]; *mood‐related outcomes*, such as depression and anxiety, measured by the Patient Health Questionnaire‐9 (PHQ‐9), the Cornell Scale for Depression in Dementia (CSDD), the Generalised Anxiety Disorder (GAD‐7), the Hospital Anxiety and Depression Scale (HADS) and the Rating Anxiety in Dementia (RAID) [17, 18, 19]; *quality of life* evaluated with instruments like the EuroQol‐5 dimensions (EQ‐5D‐5L), the Dementia Quality of Life Instrument (DEMQoL and DEMQoL‐Proxy) and the Adult Social Care Outcomes Toolkit (ASCOT) [17, 18, 40, 41]; *cognitive function* assessed using the Mini Mental State Examination (MMSE), the Large Allen Cognitive Level Screen (LACLS) and the Autobiographical Memory Interview—Extended (AMI‐E) [18, 19, 54]; *daily activities* assessed with the Pool Activity Level (PAL), the Instrumental Activities of Daily Living (IADL), the Bristol Activities of Daily Living Scale (BADLS) and the Interview for Deterioration in Daily Activities in Dementia (IDDD) [17, 18, 19, 54]; *self‐management*, including self‐efficacy and sense of competence, assessed by the General Self‐Efficacy Scale (GSE), the Self‐Management Ability Scale (SMAS) and the Sense of Competence Questionnaire (SCQ) [17, 18, 19, 54]; and finally the quality of the caregiving relationship measured using the Quality of the Caregiving Relationship (QCPR) [19]. All assessment tools were validated.

## 4 Discussion

### 4.1 Summary of Main Findings

The literature on SP for PLWD in the United Kingdom is varied and lacks focus. Studies show a wide range of participants, reflecting diversity in gender, age, dementia types, living arrangements and carer relationships. SP interventions cover a broad spectrum, including cognitive, psychosocial, physical and complementary therapies, with activities like arts, exercise, aromatherapy and acupuncture. The classification of SP interventions is inconsistent, with some acting as umbrella services while others operate independently. SP services are initiated and provided through collaborative efforts between the NHS, charities and integrated services, with referrals originating from primary care, community care, charities and including self‐ and family referrals. Connectors, such as clinical staff, multidisciplinary teams and third‐sector organisations, link PLWD to SP interventions, although terminology for these roles varies across studies. Positive outcomes include improved independence, mood, social connectedness and practical support, but challenges remain, including issues with intervention suitability, emotional impact and logistics. Overall, SP shows potential but requires more coordinated approaches and better evaluation of its benefits.

### 4.2 Comparison With Existing Literature

This review highlights that the diverse nature of SP prevents it from being standardised into a uniform ‘one size fits all’ model. Consequently, SP should be viewed as a range of practices involving multiple pathways. Although empirical research has documented the existence of such pathways [23, 73, 74], the review findings reaffirm that these pathways lack regional, cultural or procedural specificity. Inadequate reporting and ambiguities in defining SP have been identified as factors contributing to these inconsistencies [75]. Current evidence advocates redefining SP as a complex intervention model, emphasising the need to delineate its core components and contextual variables to enhance understanding and standardisation of SP pathways across different regions and cultures.

A critical factor for successful SP in dementia care, identified in this review, is the presence of a well‐resourced connector who can effectively signpost PLWD to services that meet their needs. This connector should be embedded in the community, valued by the healthcare system and properly trained and resourced, including receiving dementia‐specific training.

An example of good practice is the website and toolkit developed by the Forward with Dementia initiative, which equips social prescribers to serve PLWD better [76, 77]. This review emphasises the need for similar resources to be developed, widely distributed and tailored to specific SP pathways. Findings also indicate that outcomes for PLWD in SP extend beyond traditional health metrics, aligning with the World Health Organisation's (WHO) definition of health as encompassing physical, mental and social well‐being [78]. This review demonstrates the necessity for improved metrics to capture SP's comprehensive benefits, recognising that some valuable outcomes may be challenging to quantify.

In conclusion, this review advocates that advancing SP requires a structured model that defines its core components and contextual variables. This model can facilitate stakeholder engagement, standardise SP pathways and ensure that resources are effectively utilised to support PLWD.

### 4.3 Strengths and Limitations

This systematic review of SP for PLWD offers several key strengths. It provides a comprehensive overview of diverse SP interventions, including various activities, components and delivery modes. This diversity showcases best practices and innovative approaches, offering valuable insights for policymakers and practitioners to design person‐centred SP interventions. The inclusion of different populations across dementia stages, nationalities and regions ensures the findings are culturally relevant and adaptable to specific community needs. Additionally, input from multiple stakeholders enhances the analysis by incorporating diverse perspectives.

However, the review also faces limitations. The variability in study quality, design and evaluation metrics makes comparing outcomes challenging, limiting the ability to draw definitive conclusions. The complexity of SP interventions, often involving multiple elements and stakeholders, further complicates identifying which specific components contribute to effectiveness. Gaps in the literature, where certain populations or intervention types are underrepresented, reduce the generalisability of the findings. Additionally, potential biases in study selection and publication, along with inconsistent outcome reporting, necessitate cautious interpretation and highlight the need for further primary research.

### 4.4 Implications for Policy, Practice and Future Research

Policymakers can utilise the evidence from this review to promote the integration of SP into national dementia care strategies. The review identifies key components of successful interventions, offering guidance for resource allocation and funding towards programmes with proven benefits. By demonstrating the positive impacts of SP on both health and non‐health outcomes, this review supports policies that encourage holistic, person‐centred approaches to dementia care. Policies that foster collaboration between healthcare providers, community organisations, social services and welfare systems can be strengthened to ensure a more coordinated support network for PLWD.

In both clinical and community settings, this review offers valuable insights for improving service delivery in dementia care. Practitioners can use the findings to define SP better and tailor interventions to individual needs. The identification of barriers to participation and factors that enhance engagement can help practitioners design more accessible and appealing interventions, ultimately improving uptake and outcomes for PLWD.

Future research should focus on evaluating the long‐term effects of SP interventions, exploring the specific mechanisms through which SP operates and developing appropriate metrics for assessing its effectiveness. Research into the scalability and sustainability of successful interventions is crucial for broader implementation. Conceptualising SP as a complex intervention model, with clearly defined core components and contextual variables, could help standardise SP pathways and improve stakeholder engagement across different regions and countries. This review provides a foundation for advancing SP research and driving innovation in dementia care.

### 4.5 PPI Commentary

This commentary, provided by two PPI leads for the SPLENDID project—one living with young‐onset dementia and the other a current and former carer of family members with dementia—reaffirms many of the key findings and conclusions drawn from this CISR.

The first contributor noted that although they were offered SP, they declined due to already being well‐connected and active. In their experience working with social prescribers, challenges in rural areas were highlighted, including insufficient funding and limited transport options, which restricted access to activities. They emphasised the importance of providing support to individuals with dementia when first engaging in activities, as many ‘lost the confidence and skills they previously had’. Building trust through meaningful conversations with social prescribers was also deemed essential for encouraging participation in social or creative activities.

The second contributor, reflecting on their caring experience, stressed the need for a coordinated SP service. They argued that following a dementia diagnosis, individuals should have been directed to a single point of contact to access all necessary services throughout the dementia journey. They noted the absence of SP support during their first caregiving experience and believed that such services would have made the process ‘easier, less confusing, and with direction and purpose’. They also called for SP services to be inclusive and responsive to changing needs, with regular follow‐up and review to ensure continued support.

## Author Contributions

**Evie Papavasiliou:** conceptualisation, writing – original draft, methodology, writing – review and editing, visualisation, investigation, formal analysis, data curation, resources, project administration, supervision. **Jessica Marshall:** investigation, visualisation, writing – review and editing, formal analysis, methodology, software, data curation, resources, conceptualisation. **Louise Allan:** funding acquisition. **Katherine Bradbury:** resources. **Chris Fox:** funding acquisition. **Matthew Hawkes:** methodology, validation. **Anne Irvine:** validation, resources. **Esme Moniz‐Cook:** funding acquisition. **Aimee Pick:** resources. **Marie Polley:** funding acquisition, resources. **Amy Rathbone:** resources. **Joanne Reeve:** funding acquisition. **Dame Louise Robinson:** funding acquisition. **George Rook:** resources, validation. **Euan Sadler:** funding acquisition. **Emma Wolverson:** funding acquisition. **Sarah Walker:** investigation, visualisation, methodology, software, resources, conceptualisation. **Jane Cross:** funding acquisition, supervision, conceptualisation, methodology, project administration.

## The SPLENDID Collaboration

Prof. Chris Fox, Prof. Jane Cross, Prof. Louise Allan, Prof. Anthony Avery, Dr Katherine Bradbury, Anne Irvine, Jessica Marshall, Prof. Antonieta Medina‐Lara, Prof. Esme Moniz‐Cook, Nia Morrish, Prof. Martin Orrell, Dr Evie Papavasiliou, Aimee Pick, Prof. Fiona Poland, Dr Marie Polley, Dr Amy Rathbone, Prof. Joanne Reeve, Prof. Dame Louise Robinson, George Rook, Dr Euan Sadler, Dr Kritika Samsi, Prof. Lee Shepstone, Dr Sarah Walker and Dr Emma Wolverson.

## Conflicts of Interest

Euan Sadler declares they are a NIHR Research for Patient Benefit (RfPB) funding committee panel member.

Joanne Reeve declares they are involved in the NIHR HSDR 130247 grant named: ‘Understanding the Implementation of Link Workers in Primary Care: A Realist Evaluation to Inform Current and Future Policy’.

Louise Robinson declares they received payment/honoraria for educational resource production and lectures from Lilly UK.

## Supporting information

Suppmat.

## Data Availability

The data that support the findings of this study are available from the corresponding author upon reasonable request.
