# Supplementary material for: Reimagining Dementia Care: A Complex Intervention Systematic Review on Optimising Social Prescribing (SP) for People Living With Dementia (PLWD) in the United Kingdom
Source: Health Expect. 2025 May 12;28(3):e70289. doi: 10.1111/hex.70289 (PMC12069811; doi:10.1111/hex.70289)
Supplement: Supplementary file 1 — Suppmat. [file HEX-28-e70289-s001.docx]

**Table S1:** Search Strings Used Across Databases and Grey Literature Sources

**Database: Medline (Ovid)**

Date of search: 15 June 2023

No. of results: 3,310

| 1 exp Dementia/  2 dement*.tw.  3 alzheimer*.tw.  4 huntington.tw.  5 lewy body.tw.  6 (cognitive adj1 (decline or dysfunction or disorder*)).tw.  7 (memory adj1 (problem* or loss)).tw.  8 Creutzfeldt-Jakob disease.tw.  9 pick disease.tw.  10 or/1-9  11 ((social or community or psychosocial) adj2 (health* or medicine or consultation or prescri* or activ* or refer* or therap* or program* or interven* or care or based or project*)).tw.  12 (referral adj1 (scheme* or art or befriending or non-clinical or non-med* or information or supported or well-being or wellbeing or guided)).tw.  13 (link* adj1 (scheme* or worker* or community)).tw.  14 linkworker.tw.  15 (Navigator adj1 (patient or care or health* or community or outreach or system* or resource or peer)).tw.  16 exercise.tw.  17 art.tw. /freq=2  18 (sport* adj2 (therap* or program* or interven*)).tw.  19 swim*.tw. /freq=2  20 befriend*.tw.  21 music.tw. /freq=2  22 singing.tw.  23 nature.tw. /freq=2  24 aquatherapy.tw.  25 ecotherapy.tw.  26 gardening.tw.  27 cooking.tw. /freq=2  28 ((sensory adj2 (experience or intervention or group)) or multisensory).tw.  29 ((animal* or pet* or dog* or cat* or pony or ponies) adj2 therap*).tw.  30 conservation.tw.  31 walking.tw.  32 outdoor.tw.  33 horticultural.tw.  34 yoga.tw.  35 mindful*.tw.  36 meditat*.tw.  37 museum.tw.  38 (health advis?r* or medical advis?r* or health trainer* or wellbeing coordinator* or well-being co-ordinator*).tw.  39 (non-drug or non-pharma*).tw.  40 exp Social Support/  41 Social Interaction/  42 (home adj2 prescri*).tw.  43 healthy living.tw.  44 wellness.tw.  45 facilitator*.tw.  46 or/11-45  47 exp United Kingdom/  48 (UK or United Kingdom or England or Ireland or Irish or Scotland or Scottish or Wales or Welsh or Britain or British or NHS or national health service).tw,au,so,gc,in.  49 or/47-48  50 and/10,46,49  51 social prescri*.tw.  52 and/10,51  53 or/50,52  54 limit 53 to yr="2003 -Current"  55 exp Animals/  56 exp Humans/  57 55 not 56  58 54 not 57 |
| --- |

**Database: Embase (Ovid)**

Date of search: 15 June 2023

No. of results: 5,992

| 1 exp dementia/  2 dement*.tw.  3 alzheimer*.tw.  4 huntington.tw.  5 lewy body.tw.  6 (cognitive adj1 (decline or dysfunction or disorder*)).tw.  7 (memory adj1 (problem* or loss)).tw.  8 Creutzfeldt-Jakob disease.tw.  9 pick disease.tw.  10 or/1-9  11 ((social or community or psychosocial) adj2 (health* or medicine or consultation or prescri* or activ* or refer* or therap* or program* or interven* or care or based or project*)).tw.  12 (referral adj1 (scheme* or art or befriending or non-clinical or non-med* or information or supported or well-being or wellbeing or guided)).tw.  13 (link* adj1 (scheme* or worker* or community)).tw.  14 linkworker.tw.  15 (Navigator adj1 (patient or care or health* or community or outreach or system* or resource or peer)).tw.  16 exercise.tw.  17 art.tw. /freq=2  18 (sport* adj2 (therap* or program* or interven*)).tw.  19 swim*.tw. /freq=2  20 befriend*.tw.  21 music.tw. /freq=2  22 singing.tw.  23 nature.tw. /freq=2  24 aquatherapy.tw.  25 ecotherapy.tw.  26 gardening.tw.  27 cooking.tw. /freq=2  28 ((sensory adj2 (experience or intervention or group)) or multisensory).tw.  29 ((animal* or pet* or dog* or cat* or pony or ponies) adj2 therap*).tw.  30 conservation.tw.  31 walking.tw.  32 outdoor.tw.  33 horticultural.tw.  34 yoga.tw.  35 mindful*.tw.  36 meditat*.tw.  37 museum.tw.  38 (health advis?r* or medical advis?r* or health trainer* or wellbeing coordinator* or well-being co-ordinator*).tw.  39 (non-drug or non-pharma*).tw.  40 exp social support/  41 social interaction/  42 (home adj2 prescri*).tw.  43 healthy living.tw.  44 wellness.tw.  45 facilitator*.tw.  46 or/11-45  47 exp United Kingdom/  48 (UK or United Kingdom or England or Ireland or Irish or Scotland or Scottish or Wales or Welsh or Britain or British or NHS or national health service).tw,au,so,gc,in.  49 or/47-48  50 and/10,46,49  51 social prescri*.tw.  52 and/10,51  53 or/50,52  54 limit 53 to yr="2003 -Current"  55 exp animal/  56 exp human/  57 55 not 56  58 54 not 57 |
| --- |

**Database: PsycINFO (EBSCO)**

Date of search: 15 June 2023

No. of results: 5,530

| S52 | S48 OR S50 |
| --- | --- |
| S51 | S48 OR S50 |
| S50 | S10 AND S49 |
| S49 | "social prescri*" |
| S48 | S10 AND S46 AND S47 |
| S47 | (UK or "United Kingdom" or England or Ireland or Irish or Scotland or Scottish or Wales or Welsh or Britain or British or NHS or "national health service") |
| S46 | S11 OR S12 OR S13 OR S14 OR S15 OR S16 OR S17 OR S18 OR S19 OR S20 OR S21 OR S22 OR S23 OR S24 OR S25 OR S26 OR S27 OR S28 OR S29 OR S30 OR S31 OR S32 OR S33 OR S34 OR S35 OR S36 OR S37 OR S38 OR S39 OR S40 OR S41 OR S42 OR S43 OR S44 OR S45 |
| S45 | facilitator* |
| S44 | wellness |
| S43 | "healthy living" |
| S42 | (home N2 prescri*) |
| S41 | DE "Social Interaction" OR DE "Conflict Resolution" OR DE "Encouragement" OR DE "Interpersonal Interaction" OR DE "Interpersonal Relationships" OR DE "Nonviolence" OR DE "Parasocial Interaction" OR DE "Peace" OR DE "Personal Space" OR DE "Physical Contact" OR DE "Psychological Safety" OR DE "Social Exchange" OR DE "Teamwork" OR DE "Teasing" OR DE "Victimization" |
| S40 | DE "Social Support" OR DE "Perceived Social Support" |
| S39 | ("non-drug" or "non-pharma*") |
| S38 | ("health advis?r*" or "medical advis?r*" or "health trainer*" or "wellbeing coordinator*" or "well-being co-ordinator*") |
| S37 | museum |
| S36 | meditat* |
| S35 | mindful* |
| S34 | yoga |
| S33 | horticultural |
| S32 | outdoor |
| S31 | walking |
| S30 | conservation |
| S29 | ((animal* or pet* or dog* or cat* or pony or ponies) N2 therap*) |
| S28 | ((sensory N2 (experience or intervention or group)) or multisensory) |
| S27 | cooking |
| S26 | gardening |
| S25 | ecotherapy |
| S24 | aquatherapy |
| S23 | nature |
| S22 | singing |
| S21 | music |
| S20 | befriend* |
| S19 | swim* |
| S18 | (sport* N2 (therap* or program* or interven*)) |
| S17 | ART |
| S16 | exercise |
| S15 | (Navigator N1 (patient or care or health* or community or outreach or system* or resource or peer)) |
| S14 | linkworker |
| S13 | (link* N1 (scheme* or worker* or community)) |
| S12 | (referral N1 (scheme* or art or befriending or non-clinical or non-med* or information or supported or well-being or wellbeing or guided)) |
| S11 | ((social or community or psychosocial) N2 (health* or medicine or consultation or prescri* or activ* or refer* or therap* or program* or interven* or care or based or project*)) |
| S10 | S1 OR S2 OR S3 OR S4 OR S5 OR S6 OR S7 OR S8 OR S9 |
| S9 | "pick disease" |
| S8 | "Creutzfeldt-Jakob disease" |
| S7 | (memory N1 (problem* or loss)) |
| S6 | (cognitive N1 (decline or dysfunction or disorder*)) |
| S5 | "lewy body" |
| S4 | huntington |
| S3 | alzheimer* |
| S2 | dement* |
| S1 | DE "Dementia" OR DE "AIDS Dementia Complex" OR DE "Alzheimer's Disease" OR DE "Dementia with Lewy Bodies" OR DE "Frontotemporal Lobar Degeneration" OR DE "Presenile Dementia" OR DE "Pseudodementia" OR DE "Senile Dementia" OR DE "Vascular Dementia" |

**Database: CINAHL (EBSCO)**

Date of search: 15 June 2023

No. of results: 3,355

| S56 | S52 NOT S55 |
| --- | --- |
| S55 | S53 NOT S54 |
| S54 | (MH "Human") |
| S53 | (MH "Animals+") |
| S52 | S48 OR S50 |
| S51 | S48 OR S50 |
| S50 | S10 AND S49 |
| S49 | "social prescri*" |
| S48 | S10 AND S46 AND S47 |
| S47 | (MH "United Kingdom+") or (UK or "United Kingdom" or England or Ireland or Irish or Scotland or Scottish or Wales or Welsh or Britain or British or NHS or "national health service") |
| S46 | S11 OR S12 OR S13 OR S14 OR S15 OR S16 OR S17 OR S18 OR S19 OR S20 OR S21 OR S22 OR S23 OR S24 OR S25 OR S26 OR S27 OR S28 OR S29 OR S30 OR S31 OR S32 OR S33 OR S34 OR S35 OR S36 OR S37 OR S38 OR S39 OR S40 OR S41 OR S42 OR S43 OR S44 OR S45 |
| S45 | facilitator* |
| S44 | wellness |
| S43 | "healthy living" |
| S42 | (home N2 prescri*) |
| S41 | (MH "Interpersonal Relations+") |
| S40 | (MH "Support, Social+") |
| S39 | ("non-drug" or "non-pharma*") |
| S38 | ("health advis?r*" or "medical advis?r*" or "health trainer*" or "wellbeing coordinator*" or "well-being co-ordinator*") |
| S37 | museum |
| S36 | meditat* |
| S35 | mindful* |
| S34 | yoga |
| S33 | horticultural |
| S32 | outdoor |
| S31 | walking |
| S30 | conservation |
| S29 | ((animal* or pet* or dog* or cat* or pony or ponies) N2 therap*) |
| S28 | ((sensory N2 (experience or intervention or group)) or multisensory) |
| S27 | cooking |
| S26 | gardening |
| S25 | ecotherapy |
| S24 | aquatherapy |
| S23 | nature |
| S22 | singing |
| S21 | music |
| S20 | befriend* |
| S19 | swim* |
| S18 | (sport* N2 (therap* or program* or interven*)) |
| S17 | ART |
| S16 | exercise |
| S15 | (Navigator N1 (patient or care or health* or community or outreach or system* or resource or peer)) |
| S14 | linkworker |
| S13 | (link* N1 (scheme* or worker* or community)) |
| S12 | (referral N1 (scheme* or art or befriending or non-clinical or non-med* or information or supported or well-being or wellbeing or guided)) |
| S11 | ((social or community or psychosocial) N2 (health* or medicine or consultation or prescri* or activ* or refer* or therap* or program* or interven* or care or based or project*)) |
| S10 | S1 OR S2 OR S3 OR S4 OR S5 OR S6 OR S7 OR S8 OR S9 |
| S9 | "pick disease" |
| S8 | "Creutzfeldt-Jakob disease" |
| S7 | (memory N1 (problem* or loss)) |
| S6 | (cognitive N1 (decline or dysfunction or disorder*)) |
| S5 | "lewy body" |
| S4 | huntington |
| S3 | alzheimer* |
| S2 | dement* |
| S1 | (MH "Dementia+") |

**Database: Cochrane Database of Systematic Reviews / CENTRAL**

Date of search: 15 June 2023

No. of results: 1,382

| ID Search Hits  #1 MeSH descriptor: [Dementia] explode all trees  #2 (dement*):ti,ab,kw (Word variations have been searched)  #3 (alzheimer*):ti,ab,kw (Word variations have been searched)  #4 (huntington):ti,ab,kw (Word variations have been searched)  #5 ("lewy body"):ti,ab,kw (Word variations have been searched)  #6 ((cognitive NEAR/1 (decline or dysfunction or disorder*))):ti,ab,kw (Word variations have been searched)  #7 ((memory NEAR/1 (problem* or loss))):ti,ab,kw (Word variations have been searched)  #8 ("Creutzfeldt-Jakob disease"):ti,ab,kw (Word variations have been searched)  #9 ("pick disease"):ti,ab,kw (Word variations have been searched)  #10 #1 or #2 or #3 or #4 or #5 or #6 or #7 or #8 or #9  #11 (((social or community or psychosocial) NEAR/2 (health* or medicine or consultation or prescri* or activ* or refer* or therap* or program* or interven* or care or based or project*))):ti,ab,kw (Word variations have been searched)  #12 ((referral NEAR/1 (scheme* or art or befriending or non-clinical or non-med* or information or supported or well-being or wellbeing or guided))):ti,ab,kw (Word variations have been searched)  #13 ((link* NEAR/1 (scheme* or worker* or community))):ti,ab,kw (Word variations have been searched)  #14 (linkworker):ti,ab,kw (Word variations have been searched)  #15 ((Navigator NEAR/1 (patient or care or health* or community or outreach or system* or resource or peer))):ti,ab,kw (Word variations have been searched)  #16 (exercise):ti,ab,kw (Word variations have been searched)  #17 (art):ti,ab,kw (Word variations have been searched)  #18 ((sport* NEAR/2 (therap* or program* or interven*))):ti,ab,kw (Word variations have been searched)  #19 (swim*):ti,ab,kw (Word variations have been searched)  #20 (befriend*):ti,ab,kw (Word variations have been searched)  #21 (music):ti,ab,kw (Word variations have been searched)  #22 (singing):ti,ab,kw (Word variations have been searched)  #23 (nature):ti,ab,kw (Word variations have been searched)  #24 (aquatherapy):ti,ab,kw (Word variations have been searched)  #25 (ecotherapy):ti,ab,kw (Word variations have been searched)  #26 (gardening):ti,ab,kw (Word variations have been searched)  #27 (cooking):ti,ab,kw (Word variations have been searched)  #28 (((sensory NEAR/2 (experience or intervention or group)) or multisensory)):ti,ab,kw (Word variations have been searched)  #29 (((animal* or pet* or dog* or cat* or pony or ponies) NEAR/2 therap*)):ti,ab,kw (Word variations have been searched)  #30 (conservation):ti,ab,kw (Word variations have been searched)  #31 (walking):ti,ab,kw (Word variations have been searched)  #32 (outdoor):ti,ab,kw (Word variations have been searched)  #33 (horticultural):ti,ab,kw (Word variations have been searched)  #34 (yoga):ti,ab,kw (Word variations have been searched)  #35 (mindful*):ti,ab,kw (Word variations have been searched)  #36 (meditat*):ti,ab,kw (Word variations have been searched)  #37 (museum):ti,ab,kw (Word variations have been searched)  #38 (("health advis*" or "medical advis*" or "health trainer*" or "wellbeing coordinator*" or "well-being co-ordinator*").):ti,ab,kw (Word variations have been searched)  #39 ((non-drug or non-pharma*)):ti,ab,kw (Word variations have been searched)  #40 MeSH descriptor: [Social Support] explode all trees  #41 MeSH descriptor: [Social Interaction] this term only  #42 ((home NEAR/2 prescri*)):ti,ab,kw (Word variations have been searched)  #43 ("healthy living"):ti,ab,kw (Word variations have been searched)  #44 (wellness):ti,ab,kw (Word variations have been searched)  #45 (facilitator*):ti,ab,kw (Word variations have been searched)  #46 #11 or #12 or #13 or #14 or #15 or #16 or #17 or #18 or #19 or #20 or #21 or #22 or #23 or #24 or #25 or #26 or #27 or #28 or #29 or #30 or #31 or #32 or #33 or #34 or #35 or #36 or #37 or #38 or #39 or #40 or #41 or #42 or #43 or #44 or #45  #47 MeSH descriptor: [United Kingdom] explode all trees  #48 ((UK or United Kingdom or England or Ireland or Irish or Scotland or Scottish or Wales or Welsh or Britain or British or NHS or national health service)) (Word variations have been searched)  #49 #47 or #48  #50 #10 and #46 and #49  #51 ("social prescri*"):ti,ab,kw (Word variations have been searched)  #52 #10 and #51  #53 #50 or #52 |
| --- |

**Database: Scopus**

Date of search: 16 June 2023

No. of results: 4,033

| ( ( TITLE-ABS-KEY ( dement* ) ) OR ( TITLE-ABS-KEY ( alzheimer* ) ) OR ( TITLE-ABS-KEY ( huntington ) ) OR ( TITLE-ABS-KEY ( "lewy body" ) ) OR ( TITLE-ABS-KEY ( ( cognitive AND near/1 ( decline OR dysfunction OR disorder* ) ) ) ) OR ( TITLE-ABS-KEY ( ( memory AND near/1 ( problem* OR loss ) ) ) ) OR ( TITLE-ABS-KEY ( "Creutzfeldt-Jakob disease" ) ) OR ( TITLE-ABS-KEY ( "pick disease" ) ) ) AND ( ( ( TITLE-ABS-KEY ( outdoor ) ) OR ( TITLE-ABS-KEY ( horticultural ) ) OR ( TITLE-ABS-KEY ( yoga ) ) OR ( TITLE-ABS-KEY ( mindful* ) ) OR ( TITLE-ABS-KEY ( meditat* ) ) OR ( TITLE-ABS-KEY ( museum ) ) OR ( TITLE-ABS-KEY ( ( "health advis?r*" OR "medical advis?r*" OR "health trainer*" OR "wellbeing coordinator*" OR "well-being co-ordinator*" ) ) ) OR ( TITLE-ABS-KEY ( ( "non-drug" OR "non-pharma*" ) ) ) OR ( TITLE-ABS-KEY ( ( home AND near/2 AND prescri* ) ) ) OR ( TITLE-ABS-KEY ( "healthy living" ) ) OR ( TITLE-ABS-KEY ( wellness ) ) OR ( TITLE-ABS-KEY ( facilitator* ) ) ) OR ( ( TITLE-ABS-KEY ( singing ) ) OR ( TITLE-ABS-KEY ( nature ) ) OR ( TITLE-ABS-KEY ( aquatherapy ) ) OR ( TITLE-ABS-KEY ( ecotherapy ) ) OR ( TITLE-ABS-KEY ( gardening ) ) OR ( TITLE-ABS-KEY ( cooking ) ) OR ( TITLE-ABS-KEY ( ( ( sensory AND near/2 ( experience OR intervention OR group ) ) OR multisensory ) ) ) OR ( TITLE-ABS-KEY ( ( ( animal* OR pet* OR dog* OR cat* OR pony OR ponies ) near/2 AND therap* ) ) ) OR ( TITLE-ABS-KEY ( conservation ) ) OR ( TITLE-ABS-KEY ( walking ) ) ) OR ( ( TITLE-ABS-KEY ( music ) ) OR ( TITLE-ABS-KEY ( befriend* ) ) OR ( TITLE-ABS-KEY ( swim* ) ) OR ( TITLE-ABS-KEY ( ( sport* AND near/2 ( therap* OR program* OR interven* ) ) ) ) OR ( TITLE-ABS-KEY ( art ) ) OR ( TITLE-ABS-KEY ( exercise ) ) OR ( TITLE-ABS-KEY ( ( navigator AND near/1 ( patient OR care OR health* OR community OR outreach OR system* OR resource OR peer ) ) ) ) OR ( TITLE-ABS-KEY ( linkworker ) ) OR ( TITLE-ABS-KEY ( ( link* AND near/1 ( scheme* OR worker* OR community ) ) ) ) OR ( TITLE-ABS-KEY ( ( referral AND near/1 ( scheme* OR art OR befriending OR non-clinical OR non-med* OR information OR supported OR well-being OR wellbeing OR guided ) ) ) ) OR ( TITLE-ABS-KEY ( ( ( social OR community OR psychosocial ) near/2 ( health* OR medicine OR consultation OR prescri* OR activ* OR refer* OR therap* OR program* OR interven* OR care OR based OR project* ) ) ) ) ) ) OR ( TITLE-ABS-KEY ( dement* OR huntington OR "lewy body" OR ( cognitive AND near/1 ( decline OR dysfunction OR disorder* ) ) OR ( memory AND near/1 ( problem* OR loss ) ) OR "creutzfeldt-jakob disease" OR "pick disease" ) AND TITLE-ABS-KEY ( "social prescri*" ) )AND PUBYEAR > 2002 AND PUBYEAR < 2024 AND ( LIMIT-TO ( AFFILCOUNTRY , "United Kingdom" ) ) |
| --- |

**Grey literature**

| ((Dementia OR Alzheimer OR Huntington OR “Lewy Body” OR “cognitive decline” OR “cognitive dysfunction” OR “cognitive disorder” OR “memory problem” OR “memory loss” OR “Creutzfeldt-Jakob” OR “pick disease”) AND (((social OR community OR psychosocial) AND (health OR medicine OR consultation OR prescribe OR prescribing OR prescription OR activity OR activities OR referral OR therapy OR program OR programme)) OR ((referral) AND (scheme OR art OR befriending OR “non-clinical” OR “non-medical” OR information OR supported OR “well-being” OR wellbeing OR guided)) OR ((link) AND (scheme OR worker OR community)) OR linkworker OR ((navigator) AND (patient OR care OR health OR community OR outreach OR system OR resource OR peer)) OR exercise OR art OR ((sport) AND (therapy OR program OR programme OR intervention)) OR swim OR swimming OR befriend OR befriending OR music OR sing OR singing OR nature OR aquatherapy OR ecotherapy OR gardening OR cooking OR ((sensory) AND (experience OR intervention OR group)) OR multisensory OR ((animal OR pet OR dog OR cat OR pony OR ponies) AND (therapy)) OR conservation OR walking OR outdoor OR horticulture OR horticultural OR yoga OR mindful OR mindfulness OR meditate OR meditation OR museum OR “health advisor” OR “medical advisor” OR “health trainer” OR “wellbeing coordinator” OR “well-being co-ordinator” OR “non-drug” OR “non-pharmacological” OR “social support” OR “social interaction” OR “home prescribing” OR “home prescription” OR “healthy living” OR wellness OR facilitator) AND (UK OR “United Kingdom” OR England OR Ireland OR Irish OR Scotland OR Scottish OR Wales OR Welsh OR Britain OR British OR NHS OR “national health service”)) OR ((Dementia OR Alzheimer OR Huntington OR “Lewy Body” OR “cognitive decline” OR “cognitive dysfunction” OR “cognitive disorder” OR “memory problem” OR “memory loss” OR “Creutzfeldt-Jakob” OR “pick disease”) AND (“social prescribing” OR “social prescription”)) |
| --- |

**Table S2**: PICOTS Inclusion/Exclusion Criteria

| **PICOTS** | **Inclusion** | **Exclusion** |
| --- | --- | --- |
| **Population** | *Person living with dementia:*   - Anyone of any age, sex, ethnicity, and socio-economic status living with diagnosed dementia (of any subtype or stage). - Living in the community (residential care homes included), either independently or supported by others.   *Carers:*   - Of any age, sex, ethnicity, and socio-economic status.   Providing unpaid care help or support to a family member, partner or friend living with diagnosed dementia (of any subtype or stage) but can be receiving a carer’s allowance. | *Person living with dementia:*   - Not having a formal dementia diagnosis. - Living in a nursing home, a hospice or receiving inpatient or end of life care.   *Carers:*   - Anyone providing care to PLWD not having a formal dementia diagnosis.   Anyone providing paid care, help or support to PLWD (of any subtype or stage). |
| **Intervention** | *Social prescribing (defined as):* “a means for trusted individuals in clinical and community settings to identify that a person has non-medical, health related social needs and to subsequently connect them to non-clinical supports and services within the community by co-producing a social prescription – a non-medical prescription, to improve health and wellbeing and to strengthen community connections. [11 p.9]”  **Any non-medical interventions** (activities, groups, or services within the community that meet the practical, social, and emotional needs that affect health and wellbeing of patients living with dementia and/or their carers).  run by  **Any voluntary or community sector organisations** (either community based or virtual)  accessed through  **Any referral route** (such as primary care (GPs), charity, community interest group, local authority, self-referral, carer-referral but not limited to).  via a  **Connector** (a trusted individual in a clinical or community setting involved in the delivery of social prescribing services)  through a  **Personalised co-produced care plan** (a verbal or written holistic, person-centered adaptive plan to address non-medical health related needs based on ‘what matters’ to a person) | - Any medical interventions (e.g., pharmacological). - Any non-medical interventions accessed without a connector being involved and no personalised co-produced care plan used. |
| **Context** | *Published output:*   - Focusing on UK healthcare - Papers of any empirical design - Grey literature (reports)   January 2003-October 2023 | *Published output:*   - Focusing on International healthcare - Reviews - Case studies - Editorials - News   Conference abstracts & proceedings |
| **Outcomes** | Any quantitative (e.g., scores of psychometric instruments and/or other standardised measures) or qualitative (perceptions, thoughts, experiences) individual (health and non-health) and systemic outcomes, including proxy perspectives (see Figure 1). | No restriction |
| **Timing** | No time restriction | No time restriction |
| **Setting** | Community based living anywhere in the UK. | PLWD living in a nursing home, a hospice or receiving inpatient or end of life care. |

**Figure S1:** Process Oriented Logic model (iteration 3)

**PARTICIPANTS**

**PLWD:** Anyone of any age, sex, ethnicity and socio-economic status living with diagnosed dementia^1^ of any subtype

**Carers of PLWD^2^:** Anyone of any age, sex, ethnicity and socio-economic status who provides unpaid care, help or support to a family member, partner or friend who needs help because they have a dementia diagnosis

**Theory:**

- Aims of social prescribing^3^ for PLWD and/ or their carers
- Any implicit or explicit ideas about how social prescribing works for PLWD and/or their carers in what circumstances and why

**Design:**

**Execution:**

- Timing of social prescribing (not constrained by specific timings)
- Level of engagement with social prescribing (unlimited)
- Intensity of social prescribing (varied)
- Duration of social prescribing (varied)
- Process of social prescribing (identifier (optional), connector^4^, co-produced care plan, voluntary or community sector organisation).

**Components:**

- Types of non-medical intervention^5^: educational, social, cultural, arts, advice, physical activity, volunteering, befriending, therapy, peer support, cognitive, case -management, psychosocial, community & wellbeing, occupational & complementary services

**Delivery^6^:**

**Delivery agents:**

- Any trusted individual acting as a connector in the delivery of social prescribing services, who empowers a person through holistic support and a personalised co-produced care plan^7^ to connect them to voluntary or community sector organisations,^8^ delivered desirably by people with lived experience.

**Delivery mechanisms:**

- NHS (primary & secondary care)
- Charities
- Local authorities
- Community interest groups
- Voluntary or community sector organisations
- Open referral system (including family/self-referral)

**Setting:**

- Community based living9 in the UK.
- Variable setting influences the intervention design and delivery.

**OUTCOMES**

- Improved physical and/or mental wellbeing^10^;
- Improved quality of life^11^;
- Improved mood and well-being;
- Continuity of care;
- Enhanced Independence;
- Positive behaviour change / dementia symptom management;
- Maintaining a sense of identity.

**INTERVENTION**

**IMPLEMENTATION**

**Policy:**

NHS Long Term Plan 2019; A Plan for Scotland 2016-17; Prosperity for all 2017; Planning guidance for COVID-19 recovery; Major conditions strategy: case for change and our strategic framework 2023; Universal Personalised Care: Implementing the Comprehensive Model 2019; NHS Five year Forward View 2014; General Practice Forward View 2018

**Funding:**

Allocation of funds & funding bodies for SP projects; Lack of health budget for ageing population; Social care funding crisis.

**Organisation:**

Available facilities and resources dedicated for implementation of SP projects and connectors, Long waiting lists & ongoing strikes of professionals

**Provider:**

Any evidence relating to attitudes, communication skills, beliefs about capabilities and self-efficacy, emotions, motivation, attitudes towards intervention (multidisciplinary working; lack of commitment), knowledge, skills (lack of skills), training, and professional development of delivery agents (lack of engagement of GPs; lack of training)

**CONTEXT**

**Epidemiological:**

- Ageing population
- Increased number of PLWD
- Timing of dementia diagnoses, type and stage
- Co-morbidities

**Socio-cultural:**

- Dementia stigma, cultural, religious, and language barriers within SP projects that prevent these schemes from being diversity friendly
- Lack of healthcare trust and knowledge amongst ethnic minorities

**Geographical:**

- Infrastructure at a given location (diverse)
- Access (transport, accessibility, and timing of sessions)

**Legal:**

- Mental Capacity Act 2005
- Lasting Power of Attorney (LPA)
- Advance Statement
- Advance Decision

**Socio-economic:**

- Increasing cost/socioeconomic burden for PLWD
- Financial constraints
- Digital Divide

**Health**

**Non -Health**

- Improved capacity for daily living;
- Improved engagement;
- Improved social relationships;
- Increased sense of identity (self & community);
- Activities evoking frustration or anxiety;
- Pre-existing family dynamics affecting commitment
- Empowered individuals (having a greater say in their lives and health);
- Improved / deteriorated PLWD-carer relationship and communication;
- Increased dementia knowledge and support network;

GLOSSARY OF TERMS & DEFINITIONS*:

1. *Dementia: a diagnosed syndrome related to cognitive and behavioural decline which over time affects memory, problem solving, language, mood, motivation, and behaviour to such an extent that it interferes with a person's daily life and activities.*
2. *Carers of PLWD: Anyone of any age who provides unpaid care, help or support to a family member, partner or friend who needs help because they have a dementia diagnosis.*
3. *Social Prescribing: a means for trusted individuals in clinical and community settings to identify that a person has non-medical, health related social needs and to subsequently connect them to non-clinical supports and services within the community by co-producing a social prescription – a non-medical prescription, to improve health and wellbeing and to strengthen community connections [11 p.9].*
4. *Connector: A trusted individual in a clinical or community setting involved in the delivery of social prescribing services, who empowers a person through holistic support and a personalised co-produced care plan to connect them to voluntary and community sector organisations.*
5. *Non-medical intervention: A non-clinical approach, activity, opportunity, or support scheme.*
6. *Delivery: Intervention delivery describes the ‘how’ (delivery mechanisms), ‘who’ (delivery agents), ‘where’ (setting) of the intervention [25].*
7. *Personalised co-produced care plan: A verbal or written holistic, person-centred adaptive plan based on ‘what matters’ to a person. It is produced in equal partnership with a trusted individual in a clinical or community setting to address non-medical health related needs.*
8. *Voluntary and community sector organisations: A not for profit, non-clinical community asset or scheme that offers people in-person or remote services, activities, opportunities, or support.*
9. *Community based living: Living primarily in the community, either independently or supported by others, including in a residential care home, but not in a nursing home, a hospice or receiving inpatient or end of life care.*
10. *Wellbeing: Exists in two dimensions. Subjective wellbeing relates to how people feel and how they function on a personal level in relations to their financial, health, social, personal, and local environment. Objective wellbeing relates to objective measures of an individual’s being and assumptions about basic human needs and rights.*
11. *Quality of life: A multidimensional measure of an individual’s health in terms of (but not limited to) their physical, psychological, social, personal, and environmental state.*

*Working definitions of terms for which inconsistency and/or ambiguity persists in the current literature were specifically developed drawing on stakeholder conversations, clinical, peer reviewed and grey literature, being adapted to fit the aims of this CISR.

**Green text** = Adaptions, modifications, and additions to the initial logic model (iteration 1). These represent new evidence-based insights, components, and scope that have emerged during the review’s extraction and synthesis stage and SPLENDID CISR PPI Consultations (Round 2).

**Blue text** = Adaptions, modifications, and additions to the initial logic model (iteration 1). These represent new evidence-based insights, components, and scope that have emerged during the review’s writing up and pre-dissemination stage, and the SPLENDID CISR PPI Consultations (Round 3).
